# Angiotensin-Converting Enzyme Inhibitors to Prevent Liver Fibrosis in Metabolic Dysfunction-Associated Steatotic Liver Disease: Scientific Speculation or an Opportunity to Improve Real Clinical Practice?

**DOI:** 10.3390/ijms262411782

**Published:** 2025-12-05

**Authors:** Aurelio Seidita, Carola Buscemi, Diana Di Liberto, Mirco Pistone, Salvatore Maestri, Giorgia Cavallo, Salvatore Cosenza, Gabriele Spagnuolo, Alessandra Giuliano, Daniela Carlisi, Giovanni Pratelli, Francesca Mandreucci, Antonio Carroccio

**Affiliations:** 1 Unit of Internal Medicine, “V. Cervello” Hospital, Ospedali Riuniti “Villa Sofia-Cervello”, 90133 Palermo, Italy; aurelio.seidita@unipa.it (A.S.); carola.buscemi@unipa.it (C.B.); mirco.pistone@gmail.com (M.P.); maestrisalvatore@gmail.com (S.M.); giorgia.cavallo96@gmail.com (G.C.); toto.cosenza20@gmail.com (S.C.); gabrielespagnuolo1995@gmail.com (G.S.); francesca.mandreucci@gmail.com (F.M.); 2 Department of Health Promotion Sciences, Maternal and Infant Care, Internal Medicine and Medical Specialties (PROMISE), University of Palermo, 90127 Palermo, Italy; alegiuliano94@gmail.com; 3 Institute for Biomedical Research and Innovation (IRIB), National Research Council (CNR), 90133 Palermo, Italy; 4 Institute of Biochemistry, Department of Biomedicine, Neurosciences and Advanced Diagnostics (BIND), University of Palermo, 90127 Palermo, Italy; daniela.carlisi@unipa.it (D.C.); giovanni.pratelli@unipa.it (G.P.)

**Keywords:** renin-angiotensin-aldosterone system, angiotensin-converting enzyme inhibitors, hepatic stellate cells, autophagy, liver fibrosis

## Abstract

The role of hepatic stellate cells (HSCs) in the development of liver fibrosis and portal hypertension has already been largely clarified. Activation of HSCs might lead to self-increased proliferation and enhanced contractile activity, causing their transdifferentiation into myofibroblasts (activated HSCs), which drive the release of proinflammatory mediators, collagen, proteoglycans, and other extracellular matrix components, responsible for liver fibrosis and portal hypertension development. A possible mechanism for the pathophysiological role of HSCs in liver fibrosis might be autophagy, which breaks down the lipid droplets in quiescent HSCs, releasing fatty acids and providing the energy required for their activation into myofibroblasts. An ever-growing body of scientific evidence indicates that renin–angiotensin system (RAS) blockade can inhibit the evolution of fibrosis in patients with chronic liver diseases, and especially metabolic dysfunction-associated steatotic liver disease (MASLD), although the use of both angiotensin-converting enzyme (ACE) inhibitors and angiotensin receptor blockers (ARBs) has not yet been officially identified as a potential fibrosis treatment. More recently, researchers have shown that overexpression of ACE2, induced by ACE inhibitor (ACEI) activity and leading to the degradation of angiotensin (ANG) II into ANG 1-7, inhibition of autophagy and consequent HSC activation, might prevent liver fibrosis development. This review aims to summarize recent pre-clinical studies and to identify a common thread underlying the latest scientific evidence in this field.

## 1. Introduction

In 2023, an epidemiological investigation, conducted on the Ipsos nonalcoholic steatohepatitis (NASH) therapy monitor database regarding real clinical practice in the management of patients with NASH, highlighted that 28% of these patients were prescribed angiotensin-converting enzyme (ACE) inhibitors (ACEIs) [1].

Nevertheless, neither the American Association for the Study of Liver Diseases (AASLD) [2] nor the European Association for the Study of the Liver (EASL) [3] recommend the use of these drugs in their updated guidelines.

Considering that such a high number of patients are prescribed ACEIs, a question arises: what is the evidence and what are the purposes that lead specialists to prescribe these drugs even though they are not explicitly included in the guidelines?

According to recent evidence, inactivation and/or death of hepatic stellate cells (HSCs) via autophagy mediated by blockade of the renin–angiotensin–aldosterone system (RAAS) could reduce the degree of fibrosis [4,5]. However, most of the data regarding this potential effect of RAAS blockade have been obtained in vitro or in animal models, while evidence from studies performed in patients with Metabolic Dysfunction-Associated Steatotic Liver Disease (MASLD) is still lacking [6,7].

In this scenario, this review aims to summarize recent pre-clinical studies on ACE and liver fibrosis and to search for a common thread behind the latest scientific evidence in this field, to understand if the use of ACEIs could represent a real opportunity to prevent fibrogenesis in MASLD patients.

## 2. Methods

We performed an electronic search in the PubMed/MEDLINE, Scopus, and Web of Science for literature databases updated to September 2025. A combination of the following keywords (including MESH terms) was used: “liver steatosis” OR “NASH” OR “MASLD” OR “MAFLD” OR “MASH” OR “liver fibrosis” AND “ACE” OR “ARB” OR “angiotensin”.

After performing a first screening based on the titles and abstracts of the potential studies to assess their adherence to the current topic, the full-text articles were evaluated and used for this review, apart from articles for which the full English text was not accessible.

## 3. MASLD: Do You Remember What We’re Talking About?

It is estimated that MASLD affects 30% of the adult population worldwide, with a prevalence which increased from 22% in 1991 to 37% in 2019 [8].

Metabolic dysfunction-associated steatohepatitis (MASH) is the more severe form of MASLD. It is defined histologically by the presence of lobular inflammation and ballooning of hepatocytes and is associated with a higher risk of fibrosis progression; patients with severe liver fibrosis have an increased risk of liver-related mortality [9].

Liver fibrosis and cirrhosis are the expected outcomes of chronic liver disease regardless of etiology (alcoholic, viral or metabolic) [10].

Liver cirrhosis is a significant burden worldwide, accounting for 2 million deaths per year [11]. Even though elimination of the causative agent, e.g., eradication of the viral infection, has shown that liver fibrosis can be reversible, there is no safe approved disease-modifying therapy to treat fibrosis and prevent its principal complications in advanced chronic liver disease (ACLD): portal hypertension, variceal hemorrhage, ascites, encephalopathy, liver failure, hepatocellular carcinoma (HCC) and death [10].

Current therapies are based on the treatment of symptoms and complications. Understanding the mechanisms leading to liver fibrosis and thus identifying potential therapeutic targets for the prevention and treatment of liver fibrosis is currently a challenge.

## 4. Role of the Renin-Angiotensin System in Liver Disease: Not Just Talking About the Cardiovascular System

The renin–angiotensin–aldosterone system (RAAS) has a well-described key role in cardiovascular homeostasis. ACEIs and angiotensin receptor blockers (ARBs) are widely used in the treatment of hypertension, chronic kidney disease (CKD) and heart failure (HF), since RAAS overactivation is observed in these conditions [12].

ACEIs and ARBs have a well-established anti-remodeling effect in HF and an anti-proteinuric effect in CKD [13], and inhibition of the RAAS using ACEIs or ARBs has shown to significantly reduce morbidity and mortality due to HF [14,15].

The safety and cost-effectiveness of ACEIs and ARBs are advantageous when compared with no treatment [16].

Recent literature data suggest a possible role of the renin-angiotensin system (RAS) in liver fibrosis and cirrhosis due to inflammation and endothelial dysfunction. The functioning of the RAS is based on the balance of two main pathways: the classical and the alternative.

**Figure 1:**
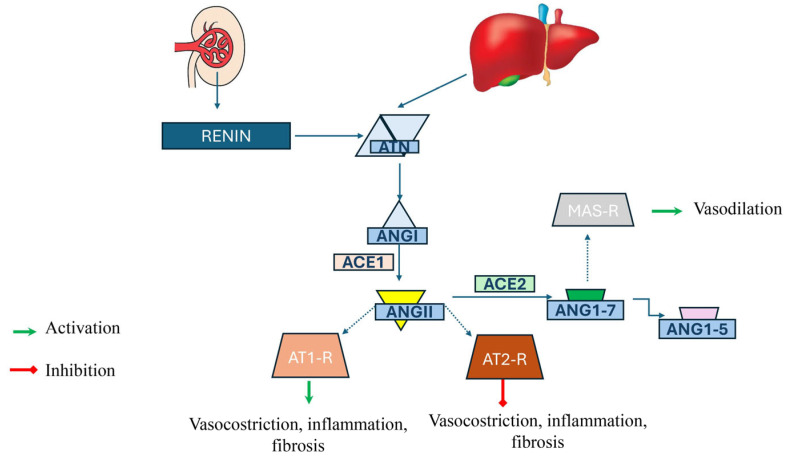
Simplified Renin Angiotensin System. Figure abbreviations: RAS: renin angiotensin system; ATN: angiotensinogen; ANG I: angiotensin I; ACE: angiotensin converting enzyme; ANG II: angiotensin II; AT1-R: angiotensin 1 receptor; AT2-R: angiotensin 2 receptor; ANG 1-7: angiotensin 1-7; ANG 1-5: angiotensin 1-5; MAS-R: MAS receptor.

In the classical pathway, angiotensinogen (ATN), secreted by the liver, is converted into angiotensin I (ANG I) by renin, which is mainly produced by juxtaglomerular cells in the kidney. ANG I is cleaved by ACE to result in angiotensin II (ANG II), which is a ligand for the angiotensin 1 receptor (AT1-R) and promotes vasoconstriction, inflammation, and fibrosis. In the alternative pathway, ANG II can also bind to the angiotensin 2 receptor (AT2-R), inhibiting vasoconstriction, inflammation, and fibrosis. Angiotensin-converting enzyme 2 (ACE 2) processes ANG II to angiotensin 1-7 (ANG 1–7) and angiotensin 1-5 (ANG 1-5) which promote vasodilation by binding to the MAS receptor (MAS-R). The two pathways are described in Figure 1.

ACE2 has recently emerged as a promising target for fibrosis treatment due to its involvement in these key signaling pathways. The proteomics study by Blomdahl et al., conducted on two cohorts of MASLD patients who underwent liver biopsy, underscored the critical role of ACE2. When included in a three-protein model, ACE2 significantly contributed to achieving the highest diagnostic performance in the non-invasive and accurate differentiation between patients with no or mild fibrosis (F0–1) and those with significant fibrosis (F2–4) [17]. Immunohistochemical analysis of liver biopsy tissue from patients with MASLD showed similar amounts of ACE2 protein in subjects with steatosis and healthy controls. Increased levels were found in patients with MASH without fibrosis [18].

Finally, ACE2 levels were similar in patients with advanced fibrosis or cirrhosis, suggesting that ACE2 might only be involved in regulating responses in the earlier stages of chronic liver disease [19].

In a recent prospective study, Hartl, L. et al. assessed the systemic and hepatic role of the RAS in ACLD; in decompensated ACLD patients plasma levels of angiotensin, ANG I, ANG II, and aldosterone, as well as ANG 1-7 and ANG 1-5 were significantly higher than in compensated ACLD patients [20]. Consistent with these results, Goh, G.B. et al. concluded that hypertensive patients with MASLD on baseline RAS blocker therapy had less advanced hepatic fibrosis, suggesting a beneficial effect of RAS blockers in MASLD [21].

One of the main objectives of recent scientific research is to identify the critical pathways leading to dysregulated angiogenesis. Under physiological conditions, angiogenesis limits fibrotic progression by increasing matrix metalloproteinase (MMP) activity. Conversely, pathological angiogenesis promotes hepatic fibrosis due to the accumulation of extracellular matrix (ECM). Recent studies have demonstrated that the main source of myofibroblasts is not only hepatic stellate cells (HSCs) but also endothelial cells (ECs) can contribute to hepatic fibrosis by activating myofibroblasts—key effectors of ECM overproduction—through the endothelial to mesenchymal transition (EndMT) [22,23]. Yes-associated protein (YAP), a transcriptional coactivator, plays a pivotal role in fibrotic progression through the induction of EndMT [24].

Y. Zhou et al. conducted a study using a murine model to investigate how ANG II influences EndMT and exacerbates hepatic fibrosis by upregulating YAP in ECs. Their findings revealed that both genetic deletion of ANG II and pharmacological intervention with olmesartan—an AT1-R (angiotensin II type 1 receptor) antagonist—effectively inhibited fibrotic progression and reduced liver damage. These results suggest that olmesartan may represent a promising therapeutic candidate for the treatment of hepatic fibrosis [25].

Furthermore, Kim, G. et al. performed a systematic review and meta-analysis of the literature and concluded that RAS blockers are potential therapeutic agents for liver fibrosis and can be safely used in patients with chronic liver disease [6].

In addition, Zhang, X. et al. affirmed in a retrospective study that ACEI intake seems to prevent liver-related events (*p* < 0.001), liver cancer (*p* = 0.002), and cirrhosis complications (*p* < 0.001) in MASLD, especially in patients with concurrent CKD (CKD-weighted subdistribution hazard ratio, 0.74; 95% CI, 0.52–0.96; *p* = 0.036; non-CKD-weighted subdistribution hazard ratio, 0.15; 95% CI, 0.07–0.33; *p* < 0.001). This same effect was not proved in the ARB treatment subgroup [7].

Even in patients with HBV infection, the use of ACEIs/ARBs appears to be associated with a reduced incidence of HCC, compared to patients who start therapy with CCBs/THZs as demonstrated in a propensity score-matched cohort study by Chen R. et al. [26].

In the meta-analysis by Chen Y. et al., which included seven studies for a total of nine cohorts, the use of RAAS inhibitors demonstrated a reduction in the risk of liver-related events (HR, 0.81; 95% CI, 0.66–1.00; *p* = 0.0493) and improved OS (HR, 0.73; 95% CI, 0.66–0.81; *p* < 0.0001). Subgroup analysis showed a percentage reduction in the development of HCC [27], data confirmed in a recent retrospective study [28].

Finally, Elhence H et al. in a recent retrospective cohort study demonstrated how the use of ACEIs/ARBs was associated with a 30% significantly lower risk of liver-related events and mortality in patients with compensated cirrhosis compared with the use of selective beta-blockers [29]. 

A summary of the main studies described above is reported in Table 1.

## 5. ACE2, Hepatic Stellate Cells and Liver Fibrosis: “A Ray of Sunshine in a Cloudy Sky”

It is well known that both liver sinusoidal endothelial cells (LSECs) and HSCs are actively involved in portal vein flow regulation by mediating intrahepatic vascular resistance [30,31,32,33].

An increasing number of studies have focused on the role of HSCs in liver fibrosis development, showing that the inactivation and/or death of activated HSCs (aHSCs) might reverse liver fibrosis [4,34,35,36,37].

More specifically, the activation of quiescent HSCs (qHSCs) leads to self-increased proliferation and contractile activity enhancement, causing them to transdifferentiate into myofibroblasts (i.e., aHSCs) able to release collagen, proteoglycans, and other extracellular matrix components, as well as proinflammatory mediators. In this light, HSC activation might determine both a reduction in hepatic sinusoidal caliber and an accumulation of extracellular matrix components, thus changing intrahepatic blood flow and structure. The study of Zhang Y et al. highlights how ANG II-mediated signaling has an impact on HSC in terms of enhanced cell contraction and production of collagen and ECM [5]. Clinical consequences of this molecular mechanism are the progression of liver fibrosis and portal hypertension, as seen from the cases in which ANG II-mediated intracellular alpha-SMA-MF-SF assembly is inhibited. These events inevitably lead to liver fibrosis and portal hypertension development [5,32,33,38].

Kuang et al. evaluated the effects of AT1-R inhibition on NASH and fibrosis in a transgenic mouse model. Compared to NASH-rats not treated with ARBs, treated rats produced fewer inflammatory cytokines and reduced galectin-3 expression, through inhibition of the TGF-β/NF-κB pathway. In vitro, ARB use reduced the expression of pPKD1 and pPKCδ proteins, thereby inhibiting NF-κB in activated HSCs [39].

According to some studies the specific mechanism responsible for the pathophysiological role of HSCs in liver fibrosis might be autophagy [40,41].

This represents a self-preserving, auto-digesting, multi-step mechanism which allows cells to ‘clean up’ damaged, useless, and potentially harmful cytosolic components and, at the same time, to respond to microbial aggression [4]. It is a metabolic process typical of all eukaryotic cells, which recognize no longer useful components, wrap them in a double-layered membrane vesicle (autophagosome) and direct them towards degradation mediated by lysosome-specific enzymes [42].

**Figure 2:**
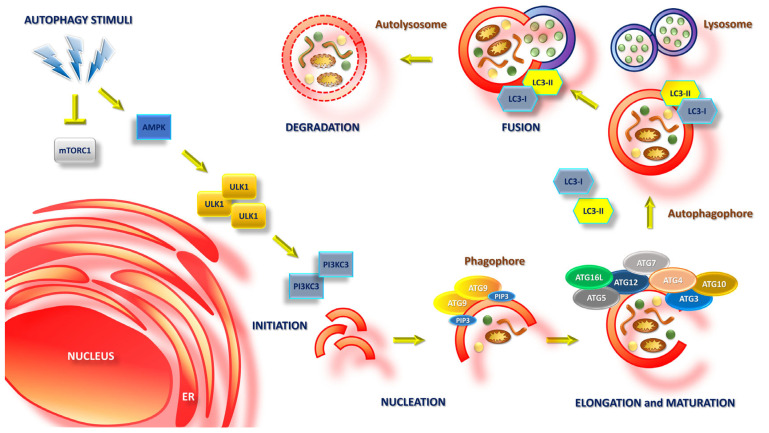
Schematic representation of the autophagy process.

This process essentially consists of four phases: (1) *initiation*, cellular stress due to an accumulation of waste products/viral infections/starvation/etc. induces the inhibition of mammalian target of rapamycin complex 1 (mTORC1) and the activation of AMP-activated protein kinase (AMPK), which leads to an overexpression of unc-51-like autophagy-activating kinase 1 (ULK1) complex; (2) *nucleation*, AMPK and the ULK1 complex mediate activation of the phosphatidylinositol 3-kinase catalytic subunit type 3 (PI3KC3) complex, in the proximity of endoplasmic reticulum regions marked by autophagy-related protein 9 (ATG9), leading to the increased production of phosphatidylinositol-3-phosphate (PI3P) and nucleation of phagophores from the endoplasmic reticulum; (3) *elongation and maturation*, increased PI3P levels cause the newly formed phagophores to interact with the ATG12-ATG5-ATG16L complex, as well as other ATG proteins (ATG3, ATG4, ATG7, ATG10), which leads to cytoplasmic microtubule-associated proteins 1A/1B light chain 3 (LC3-I) binding to the phagophores (membrane-bound-LC3, LC3-II), and, finally, to the elongation, curvature and closure of the autophagosomes; these progressively mature, shedding most of their ATG proteins and acquiring further waste material in a process mediated again by LC3-II; (4) *fusion*, mature autophagosomes fuse with lysosomes, creating the autolysosome complex in which the degradation and recycling of waste substances mediated by lysosomal enzymes occurs [43] (Figure 2).

What has just been described, known as macroautophagy, is only one of the possible ways in which the autophagy process occurs. Two others are possible: microautophagy and chaperone-mediated autophagy, which, to date, do not seem to be involved in the development of HSC-mediated liver fibrosis.

## 6. Autophagy’s Dual Role in Liver Fibrosis Development

Autophagy usually represents a fundamental mechanism for the correct homeostasis of the liver, mediating the survival and normal functionality of hepatocytes, and playing a protective role for the latter in multiple conditions such as drug-induced hepatitis, some chronic liver diseases and ischemia/reperfusion injury [43,44,45,46,47]. Similarly, it appears to play an anti-inflammatory and cytoprotective role in Kupffer cells and LSECs, preventing liver fibrosis development [48,49,50,51].

Nonetheless, in the case of HSCs the role of autophagy appears to be exactly the opposite. The first researchers to analyze this process in 2011 were Thoen, L.F. et al., who concluded their paper with the following statement: ‘During HSC activation, autophagic flux is increased. The demonstration of partial in vitro HSC activation after treatment with an autophagy inhibitor unveils a potential new therapeutic strategy for liver fibrosis’ [52]. Since then, many study groups have analyzed the relationship between autophagy induction, HSC activation and the development of liver fibrosis both on cell lines, [52,53,54,55,56,57,58,59,60,61,62,63,64,65,66,67,68,69,70,71,72,73,74,75,76,77,78,79,80,81,82,83,84,85,86] and in animal models [6,87,88,89,90,91,92,93,94,95,96,97,98,99,100] (summary in Table 2), providing a solid biological basis for this association. Unfortunately, although the biological basis for this association has been proved, it is limited to in vitro or animal model studies and, to date, no evidence is available in humans, so that studies specifically designed for this purpose are required.

However, some studies have shown that autophagy induction in HSC might protect from liver fibrosis development [101,102,103,104,105,106,107,108], thus underlining the complexity and our incomplete knowledge of these pathophysiological mechanisms. The discrepancies found between the various studies probably depend on the models that were used, with a high degree of heterogeneity in the experimental protocols (in vitro, animal, and mixed in vitro-animal models), in the markers analyzed, in the methodologies of inducing liver damage and fibrosis, and in the substances tested to modulate autophagy and fibrosis development.

**Figure 3:**
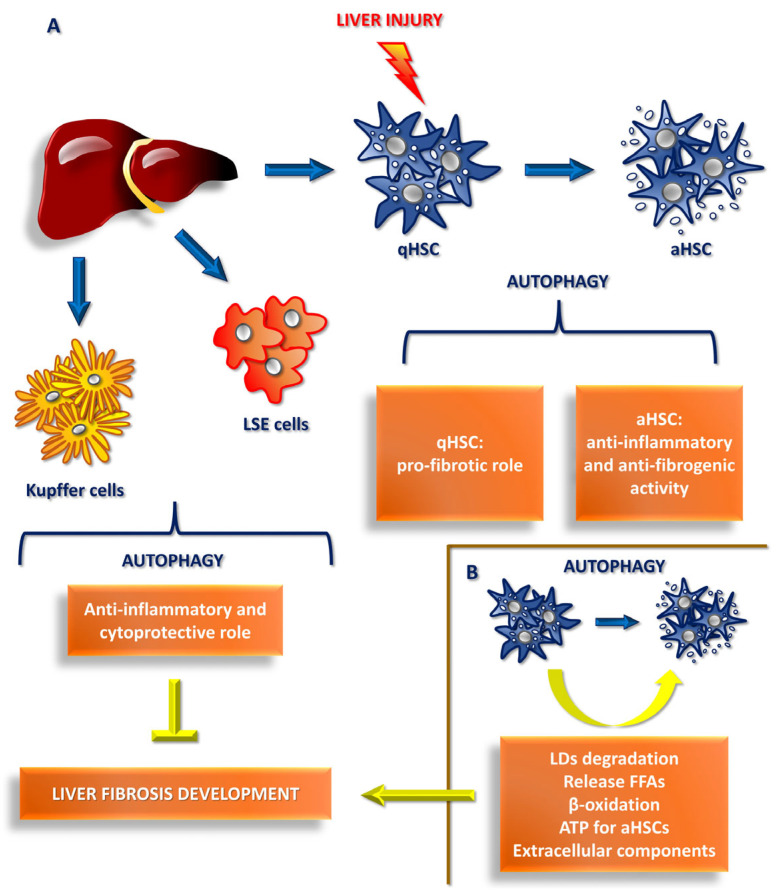
Autophagy’s dual role in liver fibrosis development. (**A**) Kupffer cell and LSEC autophagy reduces liver fibrosis development by anti-inflammatory and cytoprotective activity. (**B**) In contrast, qHSC autophagy might provide the ATP required for aHSCs to proliferate and produce ECM components inducing fibrogenesis following live injury.

Finally, it is also probable that the activation state of HSC itself could modify the results of the studies: the activation of qHSCs mediated by autophagy following liver damage could increase their profibrotic role; vice versa, if aHSCs undergo autophagy phenomena after liver damage, they could exert anti-inflammatory and anti-fibrogenic activity. In this light, drawing conclusions about the mechanisms that really underlie the autophagy-HSC activation relationship is extremely complex (Figure 3A).

In our opinion, a noteworthy hypothesis was proposed by Hernández-Gea, V. et al., according to which autophagy mechanisms in qHSCs could lead to an increase in the degradation of intracellular lipid droplets, with the release of free fatty acids, which, after beta-oxidation processes, would provide the ATP necessary for aHSCs to proliferate, produce and release the extracellular components typical of fibrogenesis [40,53] (Figure 3B).

Despite the several hypotheses that have been advanced, there is still no certainty about the role of autophagy in inducing or preventing liver fibrosis, as it is likely a very complex mechanism in which autophagy, apoptosis, ferroptosis and multiple inflammation signaling pathways interact, intersecting inextricably in a balance which, on a case-by-case basis, can lead the scales to tip towards the pro- or anti-fibrotic effect [4]. In this context, aHSCs would be induced to increase ANG II synthesis, leading, via the previously illustrated pro-fibrogenic mechanisms (Figure 1), towards fibrogenesis and, ultimately, liver cirrhosis and portal hypertension [109].

Therefore, assuming that HSCs are the liver cells which express the highest levels of ACE2 [109], it is possible to hypothesize that the key to preventing the development of fibrosis in patients with chronic liver diseases, would not be to inhibit autophagy-induced HSC activation, but rather to direct the production of ANG II in these cells, increasing ANG II—AT2R binding and/or enhancing ANG II degradation in ANG 1-7, so as to activate the anti-fibrogenic pathway.

Based on this hypothesis, a recent study analyzed the role of ACE2 in hepatic fibrogenesis and the mechanisms underlying this relationship. Wu, Y. et al. created an experimental model of liver fibrosis using 40 adult male C57BL/6J mice by intraperitoneally administering carbon tetrachloride (CCl_4_) twice a week for 8 weeks [110]. To analyze the effect of ACE 2 on liver fibrosis, the authors used a liver-specific recombinant adeno-associated viral vector (rAAV/8-ACE 2). The animals were divided into four groups (10 each): a control group not treated with CCl_4_; a group treated only with CCl_4_; a group treated with CCl_4_+rAAV/8-ACE2; and, finally, a group treated with CCl_4_+rAAV/8-ACE 2+rapamycin (a known mTOR inhibitor). Blood samples were collected to analyze several factors, including ANGII and ANG 1-7 levels, and, after sacrifice, liver samples were collected to study fibrosis degree and specific markers of both autophagy and apoptosis. The authors proved that overexpression of ACE2 (in this model induced using rAAV/8-ACE 2) increased ANG 1-7 and interleukin (IL)-10 levels, reduced ANG II levels, HSC autophagy and activation, induced HSC apoptosis and, finally, reduced the degree of liver fibrosis. These results were found only in the CCl4+rAAV/8-ACE2 group, with statistically significant differences compared to both the CCl_4_ and CCl_4_+rAAV/8-ACE2+rapamycin groups, thus suggesting the role of the AMPK/mTOR pathway in the beneficial effects induced by ACE2 [110].

Although far from offering a complete clarification, as Zhao, B.W. et al. clearly highlighted in a recent letter analyzing the paper [111], this study does propose a new and interesting perspective on the relationship between autophagy induction, HSC activation and the development of liver fibrosis, finding a common thread reconnecting the in vitro and animal model experimental evidence to findings obtained in patients treated with ACEIs or ARBs.

ACE2 can also play a role in acute liver injury (ALI). The study of Liu, L. et al. found that hyperexpression of ACE2 in transgenic mice treated with cecal ligation and puncture (CLP) was associated with a significant clinical improvement and less oxidative stress, hepatocyte apoptosis and inflammation. This effect was obtained via elevation of ANG 1-7 and MasR. Inhibition of MasR canceled the favorable effect of ACE2. The same results were obtained with bone marrow transplantation from transgenic ACE2 mice. In contrast, the ACE2 knockout group showed poor inflammatory response and more liver dysfunction. These results were consistent with the hypothesis that ACE2 protects against sepsis induced by ALI inhibiting apoptosis, reducing oxidative stress and inflammation [112].

## 7. Conclusions

An ever-growing body of scientific evidence seems to indicate that RAS blockade can positively influence the evolution of liver fibrosis in patients with chronic liver diseases, although the use of both ACEIs and ARBs has not yet been included as a potential fibrosis treatment in the guidelines of the main associations for the study of the liver (AASLD and EASL). In this scenario, the role of HSCs, activated by autophagy mechanisms, in the development of hepatic fibrosis and portal hypertension is now established.

**Figure 4:**
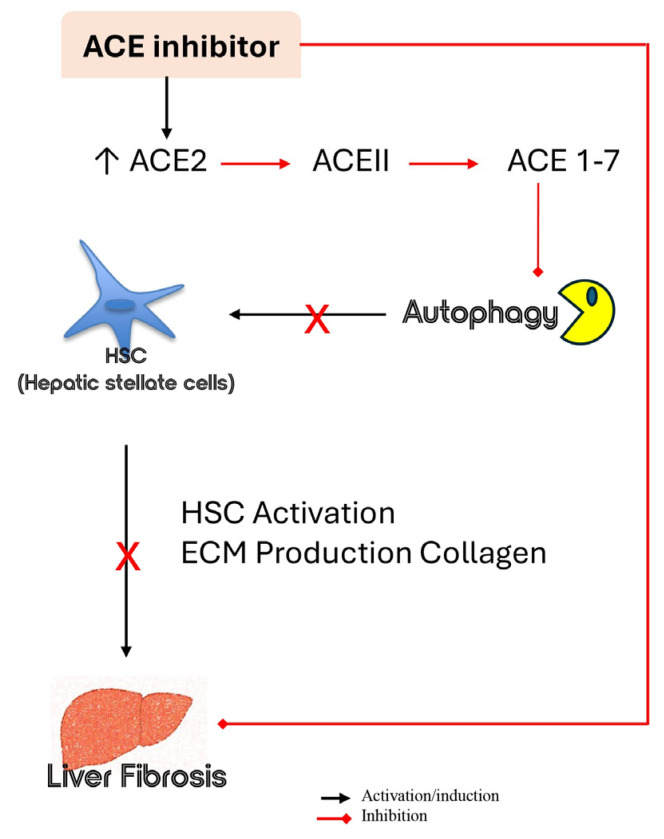
Schematic description of ACEIs function in preventing liver fibrosis.

Recent evidence has shown how the overexpression of ACE2, with the consequent degradation of ANG II into ANG 1-7 and activation of HSCs might represent the underlying pathway of fibrogenesis. This hypothesis would suggest a broader effect of ACEIs compared to ARBs in preventing liver fibrosis, even if further investigations are required as the evidence available to date does not allow definitive conclusions to be drawn (Figure 4).

Although further studies, possibly in humans, are certainly necessary to unquestionably resolve the doubts on the pathophysiological mechanisms that correlate the RAS with the development of liver fibrosis, the existing evidence certainly cannot be overlooked. Therefore, in our opinion, although not explicitly recommended by international guidelines, the use of ACEIs could represent a fascinating opportunity for preventing liver fibrosis in patients with MASLD patients, for whom the use of this category of drugs is indicated due to their antihypertensive, cardio- and nephroprotective effects.

## Figures and Tables

**Table 1:** Effects of ACEi and ARBs on liver fibrosis and liver related events.

References	Results
Zhou, Y. et al., 2024 [25]	Olmesartan may be a new generation candidate for the treatment of liver fibrosis
Kim, G. et al., 2016 [6]	The antifibrotic effects of RAS inhibitors may suggest them as a candidate therapeutic agent for hepatic fibrosis
Zhang, X. et al., 2022 [7]	ACEI, rather than ARB, treatment/treatment with ACEIs, rather than with ARBs is associated with a lower risk of liver-related events in NAFLD patients, especially among those with chronic kidney disease.
Chen, R. et al., 2024 [26]	Use of ACEIs/ARBs was associated with a reduced risk of incident hepatocellular carcinoma and liver-related deaths, compared with use of calcium channel blockers or thiazide diuretics.
Chen, Y. et al., 2025 [27]	RAS inhibitor therapy may confer dual hepatoprotective and survival benefits in patients with cirrhosis, particularly regarding hepatocellular carcinoma prevention.
Wang, R. X., et al. [28]	In patients with compensated disease, ACEIs/ARBs were not associated with hepatic decompensation or hepatocellular carcinoma.
Elhence, H., et al. [29]	ACEI/ARB use was associated with a significantly lower risk of liver related events in patients with compensated cirrhosis

Table abbreviations: ACEIs: angiotensin-converting enzyme inhibitors; ARBs: angiotensin receptor blockers; NAFLD: non-alcoholic fatty liver disease; RAS: renin-angiotensin system.

**Table 2:** Relationship between autophagy-induced HSC activation, development of liver fibrosis and potential therapeutic targets.

References	Advances in Knowledge of the Relationships Between Autophagy, HSC Activation, Liver Fibrosis and Potential Therapeutic Targets
Thoen L.F. et al., 2011 [52]	A role for autophagy during HSC activation
Hernández-Gea V. et al., 2012 [53]	Autophagy releases lipids that promote fibrogenesis by aHSCs in mice and in human tissues
Deng J. et al., 2014 [54]	HIF-1α regulates autophagy to activate HSCs
Fu M.Y. et al., 2014 [55]	TGF-β1 reduces apoptosis via autophagy activation in HSCs
He Y. et al., 2015 [56]	AGEs-induced HSC activation via autophagy contributes to hepatitis C-related fibrosis
Zhao J. et al., 2016 [57]	DMKG reduces CCl_4_-induced liver fibrosis through inhibition of autophagy in HSCs of Wistar rats
Jin Y. et al., 2016 [58]	Activation of autophagy through calcium-dependent AMPK/mTOR and PKC-θ pathway causes activation of rat HSCs under hypoxic stress
Chen M. et al., 2017 [59]	AMP-activated protein kinase regulates lipid metabolism and the fibrotic phenotype of HSCs through inhibition of autophagy
Arriola Benitez P.C. et al., 2017 [60]	*Brucella abortus* promotes a fibrotic phenotype in HSCs, with concomitant activation of the autophagy pathway
Kim K.M. et al., 2018 [61]	Gα12 overexpression induced by miR-16 dysregulation contributes to liver fibrosis by promoting autophagy in HSCs
Xie Z.Y. et al., 2018 [62]	Inhibition of autophagy reverses alcohol-induced HSCs activation through activation of Nrf2-Keap1-ARE signaling pathway
Li J. et al., 2018 [63]	Role for HMGB1 in autophagy induced HSCs activation in primary murine HSCs and human LX-2
Hong Y. et al., 2018 [64]	HMGB1-induced autophagy facilitates HSCs activation: a new pathway in liver fibrosis
Wang Y. et al., 2019 [65]	ASIC1a promotes high glucose and PDGF-induced HSCs activation by inducing autophagy
Yang R. et al., 2019 [66]	Probucol ameliorates HSCs activation and autophagy in a mouse model of liver fibrosis
Kong Y. et al., 2019 [67]	The lncRNA NEAT1/miR-29b/Atg9a axis regulates IGFBP-rP1-induced autophagy and activation of mouse HSCs
Huang T.J. et al., 2019 [68]	IGFBP-rP1 accelerates autophagy and activation of HSCs via mutual regulation between H19 and PI3K/AKT/mTOR pathway
Xie Z.Y. et al., 2019 [69]	LncRNA XIST enhances ethanol-induced HSCs autophagy and activation via miR-29b/HMGB1 axis
Guo X.H. et al., 2014 [70]	The increase in IGFBP-rP1 positively correlates with the number of collagen fibers observed
Zhang X.W. et al., 2020 [71]	Disrupting the TRIB3-SQSTM1 interaction reduces liver fibrosis by restoring autophagy and suppressing exosome-mediated HSCs activation
Seo H.Y. et al., 2020 [72]	Src inhibition attenuates liver fibrosis by preventing HSCs activation and decreasing CTGF
Hu Z. et al., 2020 [73]	TMP ameliorates hepatic fibrosis through autophagy-mediated inflammation
Wang L. et al., 2020 [74]	NK-Exo miR-223 inhibits hepatic stellate cell activation by suppressing autophagy
Wang Z.J. et al., 2021 [75]	PM_2.5_ promotes Drp-1-mediated mitophagy to induce HSCs activation and hepatic fibrosis via regulating miR-411
Li J. et al., 2021 [77]	RvD1 attenuates CCl_4_ induced liver fibrosis by inhibiting autophagy-mediated HSCs activation via AKT/mTOR pathway
Park Y.J. et al., 2021 [78]	DPX ameliorates hepatic fibrosis by inhibiting activation of HSCs through autophagy inhibition
Zheng B. et al., 2022 [76]	Autophagy of HSCs induced by *Clonorchis sinensis*
Xiao Z.H. et al., 2023 [79]	SIRT7 affects autophagy and activation of HSCs by regulating the acetylation level of HMGB1
Huan S. et al., 2023 [80]	DHA inhibits the activation and proliferation of HSCs by regulating miR-29b-3p
Le T.V. et al., 2023 [81]	Autophagy inhibitor CQ downmodulates HSCs activation and liver damage in bile-duct-ligated mice
Tan Y. et al., 2023 [82]	Berberine attenuates liver fibrosis by autophagy inhibition triggering apoptosis via the miR-30a-5p/ATG5 axis
Du J. et al., 2023 [83]	Anthocyanins improve liver fibrosis in mice by regulating the autophagic flux level of HSCs by mmu_circ_0000623
Huang F. et al., 2023 [84]	PTEN overexpression alters autophagy levels and slows sodium arsenite-induced HSCs fibrosis
Shu Y. et al., 2023 [85]	Curcumin inhibits the activity and induces apoptosis of activated HSCs by suppressing autophagy
Peng M.L. et al., 2024 [86]	Phomopsterone B alleviates liver fibrosis through mTOR-mediated autophagy and apoptosis pathway

Table abbreviations: AGEs: advanced glycation end-products; AKT: AKT protein kinase; ASIC1a: acid-sensing ion channel 1a; ATG5: autophagy-related protein 5; Atg9A: autophagy-related protein 9A; AMPK: adenosine monophosphate-activated protein kinase; CCl_4_: carbon tetrachloride; CQ: chloroquine; DHA: Decosahexaenoic acid; DMKG: dimethyl α-ketoglutarate; DPX: dendropanoxide; Drp-1: dynamin-related protein1; Gα12: guanine nucleotide-binding α-subunit 12; HIF-1α: hypoxia-inducible factor 1-alpha; HMGB1: high-mobility group box-1; HSCs: hepatic stellate cells; IGFBP-rP1: Insulin-like growth factor binding protein-related protein 1; LncRNA: long non-coding RNA; LX-2: human hepatic stellate cell lines; miR-411: microRNA-411; mTOR: mammalian target of rapamycin; NEAT1: nuclear enriched abundant transcript 1; NK-Exo miR-223: exosomal miR-223 derived from natural killer cells; PI3K: phosphatidylinositol3-kinase; PKC-θ: protein kinase C-theta; PM_2.5_: particulate matter ≤ 2.5 μm; PTEN: phosphatase and tensin homolog deleted on chromosome 10; RvD1: resolvin D1; SIRT7: sirtuin7; SQSTM1: sequestosome-1; Src: short for sarcoma; TGF-β1: transforming growth factor-beta1; TMP: tetramethylpyrazine; TRIB3: tribbles homolog 3; XIST: X-inactive specific transcript.

## Data Availability

No new data were created or analyzed in this study. Data sharing is not applicable to this article.

## Abbreviations

The following abbreviations are used in this manuscript:

AASLD	American Association for the Study of Liver Diseases
ACE	angiotensin converting enzyme
ACEIs	angiotensin converting enzyme inhibitors
ACLD	advanced chronic liver disease
aHSC	activated hepatic stellate cells
AMPK	AMP-activated protein kinase
ANG I	angiotensin i
ANG II	angiotensin II
ATN	angiotensinogen
ANG 1-5	angiotensin 1-5
ANG 1-7	angiotensin 1-7
ARBs	angiotensin receptor blockers
AT1-R	angiotensin 1 receptor
AT2-R	angiotensin 2 receptor
ATG	autophagy-related protein
CCl4	carbon tetrachloride
CKD	chronic kidney disease
EASL	European Association for the Study of the Liver
HCC	hepatocellular carcinoma
HF	heart failure
HSC	hepatic stellate cell
aHSC	activated hepatic stellate cell
qHSC	quiescent hepatic stellate cell
IL	interleukin
LC3-I	cytoplasmic microtubule-associated proteins 1A/1B light chain 3
LC3-II	membrane-bound microtubule-associated proteins 1A/1B light chain 3
MASLD	metabolic dysfunction-associated steatotic liver disease
MASH	metabolic associated steatohepatitis
MAS-R	MAS receptor
mTOR	mammalian target of rapamycin
mTORC1	mammalian target of rapamycin complex 1
NASH	non-alcoholic steatohepatitis
PI3KC3	phosphatidylinositol 3-kinase catalytic subunit type 3
PI3P	phosphatidylinositol-3-phosphate
rAAV	recombinant adeno-associated viral vector
RAA	renin-angiotensi-aldosterone system
RAS	renin-angiotensin system
ULK1	Unc-51-like autophagy-activating kinase 1
